# Prediction of Gastric Cancer Development by Serum Pepsinogen Test and *Helicobacter pylori* Seropositivity in Eastern Asians: A Systematic Review and Meta-Analysis

**DOI:** 10.1371/journal.pone.0109783

**Published:** 2014-10-14

**Authors:** Teruhiko Terasawa, Hiroshi Nishida, Katsuaki Kato, Isao Miyashiro, Takaki Yoshikawa, Reo Takaku, Chisato Hamashima

**Affiliations:** 1 Section of General Internal Medicine, Department of Emergency and General Internal Medicine, Fujita Health University School of Medicine, Toyoake, Aichi, Japan; 2 Center for Clinical Evidence Synthesis, Institute for Clinical Research and Health Policy Studies, Tufts Medical Center, Boston, Massachusetts, United States of America; 3 Department of Health Information and Statistics, Panasonic Health Care Center, Osaka, Japan; 4 Cancer Detection Center, Miyagi Cancer Society, Sendai, Miyagi, Japan; 5 Department of Surgery, Osaka Medical Center for Cancer and Cardiovascular Diseases, Osaka, Japan; 6 Department of Gastrointestinal Surgery, Kanagawa Cancer Center, Kanagawa, Japan; 7 Institute for Health Economics and Policy, Tokyo, Japan; 8 Cancer Screening Assessment and Management Division, Research Center for Cancer Prevention and Screening, National Cancer Center, Tokyo, Japan; Sapporo Medical University, Japan

## Abstract

**Background:**

To identify high-risk groups for gastric cancer in presumptively healthy populations, several studies have investigated the predictive ability of the pepsinogen test, *H. Pylori* antibodies, and a risk-prediction model based on these two tests. To investigate whether these tests accurately predict gastric cancer development, we conducted a systematic review and meta-analysis.

**Methods:**

PubMed and other electronic databases were searched for cohort studies published in English or Japanese from January 1985 through December 2013. Six reviewers identified eligible studies, and at least two investigators extracted data on population and study-design characteristics, quality items, and outcomes of interest. Meta-analyses were performed on non-overlapping studies.

**Results:**

Nine prospective cohorts from Eastern Asia reported in 12 publications, including 33,741 asymptomatic middle-aged participants of gastric cancer screening, were eligible. For discriminating between asymptomatic adults at high and low risk of gastric cancer, the pepsinogen test (summary hazard ratio [HR], 3.5; 95% confidence interval [CI], 2.7–4.7; *I^2^* = 0%) and *H. pylori* antibodies (summary HR, 3.2; 95% CI, 2.0–5.2; *I^2^* = 0%) were statistically significant predictors as standalone tests. Although the risk-prediction model was in general moderately accurate in separating asymptomatic adults into four risk groups (summary *c*-statistic, 0.71; 95% CI: 0.68–0.73; *I^2^* = 7%), calibration seemed to be poor. The study validity was generally limited.

**Conclusions:**

The serum pepsinogen test, *H. pylori* antibodies, and the four-risk-group model for predicting gastric cancer development seem to have the potential to stratify middle-aged presumptively healthy adults. Future research needs to focus on comparative studies to evaluate the impact of screening programs adopting these tests. Also, validation, preferably with model updating, is necessary to see whether the current model performance is transferable to different populations.

## Introduction

Gastric cancer is the fourth most common cause of cancer death worldwide [1], and is the most prevalent cancer in Eastern Asia [2]. Because high cure rates can be expected for early stages of gastric cancer, and non-randomized evidence suggests that radiographic screening can decrease gastric-cancer-specific mortality [3], several Asian countries have initiated cancer-screening programs using upper gastrointestinal tract photofluorography or gastric endoscopy [4]. However, recent nationwide gastric cancer screening rates for the general population in Japan have been unsatisfactorily low [5]; therefore, a major current focus is on developing a risk-stratified screening program by efficiently identifying high-risk populations.

Infection with *Helicobacter pylori* and its associated chronic atrophic gastritis (CAG) are two major risk factors for gastric cancer [6], [7]. In addition to several candidate oncogenic mechanisms [7], [8], epidemiologic studies [9]–[12], have shown the associations between these factors and gastric cancer. To predict gastric cancer development in healthy populations, several cohort studies have assessed the serum pepsinogen test and *H. pylori* seropositivity, respectively, as surrogate markers for CAG and *H. pylori* infection, and a risk-prediction model based on the two tests. However, these studies have small sample sizes and use heterogeneous designs, making it difficult to interpret the published data. Also, those studies that have assessed the prediction model typically focus on relative risk estimates and fail to assess the performance of the model [13]. Therefore, we performed a systematic review to provide a comprehensive summary of the predictive ability of these tests in presumptively healthy adults. We also aimed to quantitatively explore the calibration and discrimination of the prediction model based on the reported data.

## Materials and Methods

This work is an updated, in-depth systematic review and meta-analysis based on a broad health technology assessment conducted by the literature review committee for the Japanese Guidelines for Gastric Cancer Screening [3], using a set of standardized systematic review methods [14] and following a prespecified protocol. There is no specific protocol for this focused, updated review. The aim of the health technology assessment was twofold: in an asymptomatic healthy population, to evaluate the existing evidence on benefits and harms of conventional screening strategies using photofluorography or gastrointestinal endoscopy, and to evaluate “risk-stratified” screening strategies incorporating the serum pepsinogen test, *H. pylori* antibodies, or a risk-prediction model based on the two tests as the primary screening modality before performing photofluorography or endoscopy. In this paper, we focus on the predictive ability of the serum pepsinogen test, *H. pylori* serology, and the prediction model to predict gastric cancer development in asymptomatic populations.

### Literature search

We searched PubMed, Web of Science, Cochrane Central, and the Japanese Medical Research Database (Igaku-Chuo-Zasshi) using search terms like “stomach neoplasms”, “gastric cancer”, “endoscopy”, “*Helicobacter pylori*”, “pepsinogens”, “atrophy”, “diagnosis”, “mass screening” and their synonyms. The searches were limited to English- or Japanese-language publications, and citations from Jan 1 1985 to July 10 2013. The exact search strategy is reported in the guidelines [3]. The search was updated to December 31 2013 to include only studies assessing the serum pepsinogen test and/or *H. pylori* serology. The updated search was then supplemented by examining the title and abstract of all articles that cited at least one of the already included publications found through the citation-tracking function of the ISI Web of Knowledge database, Scopus, and Google Scholar. We also perused the reference list of eligible studies and relevant review articles, and consulted with experts in gastric cancer screening.

### Study eligibility

Six reviewers in three pairs independently screened non-overlapping sets of abstracts and independently examined the full text of each potentially eligible study. Studies that assessed the serum pepsinogen test and/or *H. pylori* seropositivity at enrollment as predictors of gastric cancer development in asymptomatic participants of gastric cancer screening programs were considered eligible. We included both prospective cohort studies and retrospective analyses of prospective cohorts of any sample size that followed up all participants. We did not prespecify a minimum follow-up period, how the studies followed up participants, or how they verified gastric cancer development. We accepted studies regardless of whether they included or excluded participants with gastric cancer diagnosed at enrollment or shortly after positive screening results for pepsinogen test and/or *H. pylori* antibodies (endoscopy and biopsy were typically performed). We excluded case-control studies and nested case-control or case-cohort studies. We also excluded studies that assessed the detection rates of gastric cancer based on the pepsinogen test and/or *H. pylori* antibodies without follow-up. Discrepancies regarding inclusion were resolved by consensus between the assessors including a third reviewer.

We took particular care to identify publications with at least partially overlapping populations by comparing authors, centers, recruitment periods, and patient demographic characteristics. In the case of multiple publications from one study, we included only the publication with the longest follow-up.

### Data extraction

One reviewer extracted descriptive data from each eligible paper, which were confirmed by at least one other reviewer. We extracted the following information: first author, year of publication, study location, study design and setting, inclusion and exclusion criteria, baseline participant demographic characteristics, follow-up period, methods used to ascertain gastric cancer development, and technical specification of the pepsinogen test and *H. pylori* antibodies. We also recorded the reported performance of each test for diagnosing respective target clinical conditions and their reference standard, if any, in the literature (i.e., CAG by pepsinogen test and *H. pylori* infection status by seropositivity).

One reviewer extracted numerical data regarding test results and gastric cancer development from each study, which were confirmed by at least one other reviewer. Specifically, for each risk group defined we recorded the cumulative number of gastric cancer cases identified through follow-up, the total number at risk, and the hazard ratio (HR) estimates from the full statistical model that adjusted for the largest number of potential confounders. Two out of 150 (1%) extractions by the second reviewer for the numerical data were inconsistent.

Any disagreements were resolved by consensus. A third investigator adjudicated any unresolved discrepancies. We contacted by email authors of studies for additional information when it was not possible to extract numerical data from the publication.

### Quality assessment

We abstracted information on aspects of the design and conduct of individual studies using a checklist specifically designed for assessing studies of prognostic tests [15]. Items included study design, selection of study participants, description of tested population, inclusion and exclusion criteria, start point of follow-up, description of test characteristics (assay methods and blinding of test assessors to clinical outcomes and *vice versa*), description of ascertainment of gastric cancer development, follow-up period, and methods of data analysis (internal and external validation, and whether appropriate statistical analyses including multivariable adjustment taking account of other established risk factors had been performed). We then judged the risk of bias for studies that assessed the pepsinogen test or *H. pylori* antibodies as a standalone test, using the Quality In Prognosis Studies (QUIPS-2) [16], and rated the risk of bias and concerns about applicability for studies of a risk-prediction model based on the two tests, using the Prediction Study Risk of Bias Assessment tool (PROBAST) [17]. One reviewer assessed study quality, and the rating was confirmed by at least one other reviewer. Three out of 64 (5%) quality ratings by the second reviewer were inconsistent. Any discrepant results were resolved by consensus.

### Data synthesis and analysis

The predictive ability of the pepsinogen test and *H. pylori* antibodies as standalone tests were analyzed using the DerSimonian-Laird random effects model meta-analysis to obtain summary HRs with their corresponding 95% confidence intervals (CIs) for studies that reported time-to-event data in the main analysis and the Mantel-Haenszel fixed-effects model meta-analysis for sensitivity analyses. For studies that reported cumulative count data, we performed the Mantel-Haenszel fixed-effects meta-analysis to obtain summary odds ratios (ORs) with their corresponding 95% CIs in the main analysis because studies in general reported the incidence rates of gastric cancer in the test-negative group to be less than 1% with substantial imbalances between the test-positive and -negative groups [18]. The Peto OR method and the Mantel-Haenszel fixed-effects model for combining summary risk differences were also used in sensitivity analyses. To supplement the measures of predictive ability, we also obtained summary estimates of sensitivity and specificity with their corresponding 95% CIs using bivariate random effects meta-analysis with the exact binomial likelihood [19] and constructed summary receiver-operating characteristic (ROC) curves and confidence regions for summary sensitivity and specificity [20].

Studies that assessed the risk prediction model based on the pepsinogen test and *H. pylori* serology consistently defined four risk groups (**Table 1**). Suboptimal methodology and reporting of model performance are common in prognostic model studies using time-to-event data [21], [22]. After perusal of the reported measures of model performance, we determined to quantitatively synthesize HRs across risk groups; no studies reported the recommended standard measures of discrimination or calibration [22]. From four risk strata, it is possible to form six pairwise comparisons. None of the studies, however, assessed and reported all the logically comparable contrasts but typically reported only three HRs of gastric cancer development, comparing Groups B, C, and D with Group A only. Therefore, in addition to conventional meta-analysis of direct evidence on the reported contrasts, we performed multivariate meta-analysis for predictive tests with three or more risk strata with a Bayesian framework to combine the totality of direct and indirect evidence in a single analysis, taking correlations between the risk strata into account [23], [24]. We calculated the summary HRs and ORs (for cumulative count data) with their corresponding 95% credible intervals (CrIs) using the fixed-effects model in the main analysis and the random-effect model in sensitivity analysis. Additionally, we calculated the probability for each risk group that it would be ranked from best to worst among the four risk strata. Finally, we repeated the multivariate meta-analysis in a *post-hoc* set of sensitivity analyses by combining Group C and Group D to form a 3-risk group model (**Table 1**).

**Table 1:** Gastric cancer risk groups defined by the pepsinogen test and *H. pylori* antibody.

Pepsinogen test	*Negative*	*Negative*	*Positive*	*Positive*
*H. pylori* serology	Negative	Positive	Positive	Negative
4-risk group model^a^	Group A	Group B	Group C	Group D
3-risk group model^b^	Group A	Group B	Group C + Group D	

a The original model adopted in the primary studies.

b An alternative model used in our *post-hoc* sensitivity analysis.

To quantitatively explore model performance with reported cumulative count data, we performed “descriptive” meta-analysis of the discrimination and calibration using the DerSimonian-Laird random-effects model [25], acknowledging not taking account of potential effects of censoring. For each study, as the measure of discrimination, we estimated the *c*-statistic and its corresponding 95% CIs [26]. To assess the calibration of the model, for each study we calculated the expected over observed event ratio (E/O) and its Poisson exact 95% CIs for each risk group and for all the risk groups combined. Expected events were calculated by applying the proportionate cumulative gastric cancer incidence estimates from long-term follow-up results of the first reported study [27] to the corresponding four risk groups of the subsequent studies assuming a constant incidence rate as reported [27]. E/O statistics less than, equal to, and more than 1 respectively suggest an under-, perfect-, and over-prediction of the model.

We quantified between-study heterogeneity with the *I^2^* statistic and considered *I^2^* to be suggestive of intermediate or high heterogeneity when >50% or >75%, respectively [28]. For each model in the Bayesian multivariate meta-analysis we based results on 3 different chains and 200,000 iterations after a burn-in of 10,000 iterations, and model convergence was assessed by Brooks-Gelman-Rubin criteria [29]. We did not perform tests for funnel plot asymmetry because there were fewer than ten eligible studies [30]. Also, we did not perform subgroup or meta-regression analyses due to the small number of studies. All analyses were conducted using Stata SE, version 12.1 (Stata Corp, College Station, TX, USA) and WinBUGS 1.4.3 (MRC Biostatistics Unit, Cambridge, UK). P*-*values for all comparisons were 2-tailed, and statistical significance was defined as a p*-*value less than 0.05.

## Results

### Literature flow and eligible studies

**Figure 1:**
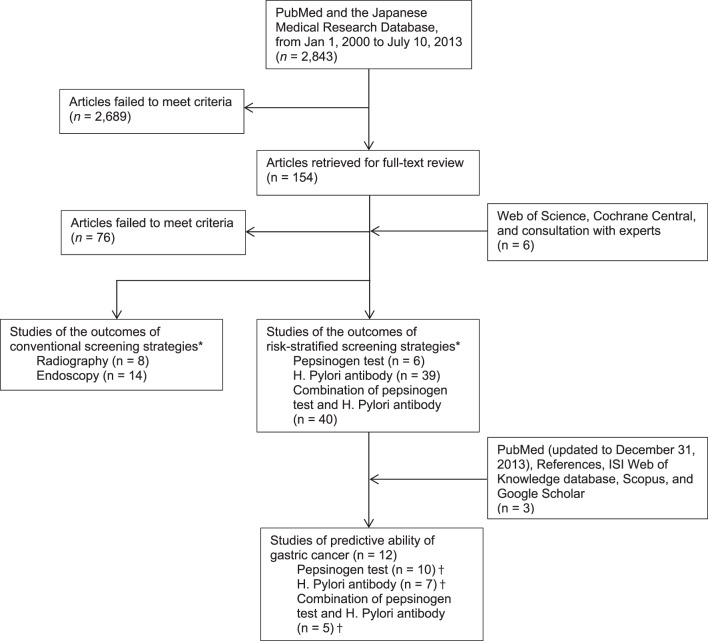
Study flow diagram. *, † These studies are not necessarily mutually exclusive; some met more than two research questions in the original health technology assessment.

Our main literature searches identified 2843 citations, of which 154 were considered potentially eligible and reviewed in full (**Figure 1**). Six additional citations were identified through supplementary searches. We excluded 76 studies that did not meet our inclusion criteria. The updated search found three additional eligible studies. In the end, 9 independent cohorts reported in 12 publications [27], [31]–[41] were considered eligible.

### Study and clinical characteristics

The 9 eligible cohort studies (7 from Japan, 1 from Korea, and 1 from China) included 33,741 asymptomatic participants of gastric cancer screening programs (**Table 2**). Five studies [32], [34], [37], [39], [40] were conducted in communities, whereas two [35], [38] were opportunistic screening in clinical settings, and another two [27], [41] were workplace health checkups. Although all studies prospectively enrolled participants, two studies [37], [39] reported that data were analyzed retrospectively. The mean age at enrollment ranged between 45 and 57 years, and the mean follow-up ranged between 3.9 and 14 years. During the study period, only between 2 and 89 gastric cancer cases were detected per cohort, which corresponded to heterogeneous cancer incidence rates of between 21 and 260 cases per 100,000 person-years. Only did 2 cohorts [27], [35] analyze gastric cancer incidence by histological subtype (i.e., intestinal type or diffuse type). Two studies excluded from the analysis cases of gastric cancer diagnosed early after enrollment: 8 cases diagnosed within 1 year in one [27], [33] and 3 cases diagnosed within 6 months in the other [41]. Review of the registry data on annual health checkups with radiographic screening and medical records was the most commonly adopted method to ascertain gastric cancer cases. Only in two studies [35], [38] was periodic endoscopic screening performed to detect gastric cancer.

**Table 2:** Study characteristics.

Study ID(Studylocation)	Design;Recruitmentperiod (*y*/*m*)	Setting	Exclusions	Subjects,*n;*(Male, *%)*	Meanage(range), *y*	Meanfollowup, *y*	Cancer incidencerate, /*100,000* *person-year*	Ascertainment ofgastric cancerdevelopment
Katsushika study[31], [32] (Katsushika,Tokyo, Japan)	Prospective;2000	Population-basedhealthcheckup	ND	4,490 (37)	47 (40–55)	3.9	46	Gastric cancer screening program registry and hospital record. Endoscopy recommended if PG test positive^a^ or barium X-ray if negative.
Wakayama study[27], [33] (Wakayama,Wakayama,Japan)	Prospective;1994/4-1995/3	Workplacehealth checkup	Women; symptomaticpatients; previousgastric resection;users of H2RAs orNSAIDs; gastriccancer diagnosed<1 year aftersurveillance (n = 8)	4,655^b^ (100)	50 (40–59)	11.6^b^	161	Annual double-contrast barium X-ray and PG test followed by endoscopy +/− biopsy if either test positive
Watase 2004[34] (Adachi,Tokyo, Japan)	Prospective;1996	Population-basedhealthcheckup	Symptomatic patients;previous gastricresection; usersof PPIs, patientswith renal failure	5,449 (37)	51 (40–60)	4.8	58	Review of health checkup database and gastric cancer screening program registry. Endoscopy recommended if positive for PG test^c^
Watabe 2005[35] (Chiba,Japan)	Prospective;1995/3-1997/2	Opportunistichealthcheckup	Gastric cancer; pepticulcer; and pasthistory of gastrectomy	6,983 (68)	49 (ND)	4.7	130	Annual endoscopy (mean 5.1 times during the follow-up period)
Hisayama study[36], [37] (Hisayama,Fukuoka, Japan)	Retrospectiveanalysisof a prospectivecohort; 1988	Population-basedhealthcheckup	Previous gastrectomyor gastric cancer;unavailable serumsample	2,446 (42)	57 (40-)	14^d^	260^e^	Records on annual health checkup and screening barium radiography; contact by mail or telephone; use of a daily monitoring system; hospital or clinic records on barium radiography, upper endoscopy, and histologic diagnosis; autopsies of subjects who died during the study period^f^.
Kim 2008 [38](Seoul, South Korea)	Prospective;1992–1998	Opportunistichealth checkup	ND	975 (90)	45 (ND)	9.9	21	Endoscopy every 1 to 3 years
Mizuno 2010 [39](Kyoto, Japan)	Retrospectiveanalysisof a prospectivecohort; 1987	Population-basedhealthcheckup	ND	2,859 (35)	ND (35-)^g^	9.3^h^	229^i^	Cancer registry based on notification by local hospitals, gastric cancer screening, activities of public health nurses, and death certificates.
Zhang 2012[40] (Zanhuang,Hebei, China)	Prospective;1996–1997	Population-basedhealthcheckup	Gastric cancer; pepticulcer; other severediseases; and subjectswith questionable *H.* *pylori* antibody results	1,501 (37)	45 (30-)	14	124^J^	Annual home visits and review of histology and X-rays from the local clinics and hospitals.
Okuno 2012[41] (Kurobe,Toyama, Japan)	Prospective;1995	Workplacehealthcheckup	Age ≥60; Previousgastric cancer; gastriccancer <6 months afterPG test (n = 3); noPG test results	4,383 (65)	45 (35–60)	12.3	111	Annual screening x-ray gastrography and/or endoscopy^k^. Self-report or physicians’ report of gastric cancer confirmed through the correspondences with the testing institutions.

a Recommendation of endoscopy with biannual follow-up contact was offered if PG test positive.

b 5,209 subjects with a mean follow-up of 9.7 years for the analysis of PG test only.

c Recommendation of endoscopy was offered annually for two years.

d 10 years for the analysis of a 4-group risk model based on both PG test and H. pylori infection status.

e Approximately estimated based on 89 gastric cancer cases identified during the follow-up period of 14 years.

f Autopsy was performed 75% of all deaths from any causes.

g 83% of participants were 74 years of age or younger.

h Median.

i Approximately estimated based on 61 gastric cancer cases identified during the median follow-up period of 9.3 years.

j Approximately estimated based on 26 gastric cancer cases identified during the followup period of 14 years.

k Total screening rates by x-ray gastrography and/or endoscopy were 78% in 1995, 71% in 1999, 75% in 2004, and 82% in 2009.

FY = fiscal year; H2RAs = histamine receptor 2 antagonist; ND = no data; NSAIDs = non-steroidal anti-inflammatory drugs; PG = pepsinogen; PPI = proton pomp inhibitor.

Three studies [32], [34], [41] evaluated the serum pepsinogen test alone, while a single study [38] exclusively assessed *H. pylori* antibodies as a standalone risk factor (**Table S1**). Five studies [27], [35], [37], [39], [40] evaluated both tests and the risk-prediction model, consisting of four risk strata based on the two tests. Of the seven studies that reported when samples were assayed, two analyzed the stored serum 7 to 14 years after blood collection. All seven studies that reported the method used to measure pepsinogen concentrations used an identical assay with a set of recommended cutoff values to diagnose CAG (pepsinogen I≤70 ng/mL and pepsinogen I/II≤3.0) [42]. Two studies adopted additional sets of cutoffs (**Table S1**). Various assays were used for *H. pylori* antibodies and heterogeneous estimates of sensitivity and specificity were reported (**Table S1**).

### Assessment of study quality

**Figure S1** shows the results of validity rating. No study adequately reported all seven items relevant to study validity that we assessed, that is, study design, selection of participants, participant characteristics, start of follow-up, test characteristics, methods of ascertainment of gastric cancer development, and methods of data analysis (**Table S2**). Reporting was particularly poor regarding blinding of interpreters of the two tests to clinical outcomes, and blinding of outcome assessors to the test results. Three studies [31], [32], [34], [39] excluded more than 50% of all potentially eligible participants, and a retrospective design was adopted in 2 studies [36], [37], [39]. The follow-up period is shorter than 5 years in three studies [31], [32], [34], [35]. Four studies [31], [32], [34], [38], [40] failed to adjust for any potential confounders in analyzing risk estimates.

### Pepsinogen test and *H. pylori* antibodies

Four studies, including 14,343 subjects [33], [37], [39], [41], reported HRs for the pepsinogen test to predict gastric cancer development. All studies but one [37] adopted the recommended cutoff values for this analysis. The random-effects meta-analysis showed that subjects with a positive test had a higher risk of gastric cancer than those with a negative test (summary HR, 3.5; 95% CI, 2.7–4.7; p<0.001; *I^2^* = 0%) (**Figure S2-A**). Cumulative count data were available in 8 studies including 32,766 subjects [27], [32], [34]–[36], [39]–[41]: a positive test result was similarly significantly associated with a higher risk of gastric cancer compared with a negative result (fixed-effects OR, 3.9; 95% CI, 3.2–4.8; p<0.001; *I^2^* = 37%) (**Figure S2-B**). These studies had a summary sensitivity of 0.57 (95% CI, 0.49–0.65) and a summary specificity of 0.76 (95% CI, 0.69–0.81) (**Figure S2-C**).

For *H. pylori* antibodies, HR estimates were available from 3 studies including 9960 subjects [33], [36], [39]. The random-effects meta-analysis showed that subjects positive for *H. pylori* antibodies had a higher risk of gastric cancer than those with a negative test (summary HR, 3.2; 95% CI, 2.0–5.2; p<0.001; *I^2^* = 0%) (**Figure S3-A**). Six studies including 19,419 subjects [27], [35], [37]–[40] reported cumulative count data for OR estimation, and the fixed-effects meta-analysis found a similarly significant association between positive *H. pylori* antibodies and a higher incidence of gastric cancer (summary OR, 2.7; 95% CI, 2.0–3.8; p<0.001; *I^2^* = 10%) (**Figure S3-B**). Summary estimates of prognostic accuracy were 0.87 (95% CI, 0.76–0.94) for sensitivity and 0.30 (95% CI, 0.23–0.39) for specificity (**Figure S3-C**).

In the preplanned sensitivity analyses for these two tests, the summary estimates of the alternative models were not materially different from those in the main analysis (data not shown).

### Risk prediction model

Predictive ability of the risk-prediction model based on the pepsinogen test and *H. pylori* antibodies was first reported in the Wakayama study of 2004 [33], where the baseline gastric cancer risk was estimated in a male population from a workplace health checkup. Four subsequent studies evaluated the model in three community-dwelling populations [35], [37], [40] and in a cohort of participants in opportunistic health checkups [39], which we considered validation cohorts.

**Figure 2:**
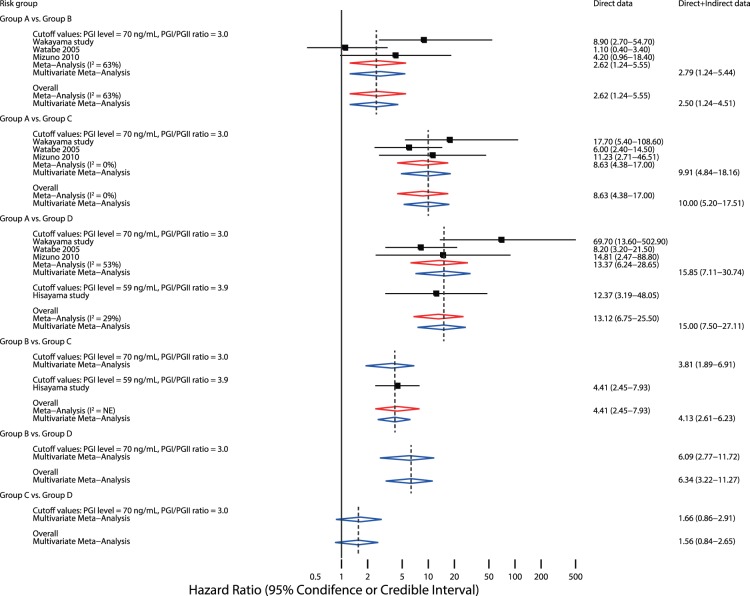
Meta-analysis of hazard ratio for four-risk-group prediction model to predict gastric cancer development. The red and blue diamonds depict a summary hazard ratio with extending 95% confidence interval (CI) or 95% credible interval (CrI), estimated from direct meta-analysis or multivariate meta-analysis, respectively. Each square and horizontal line indicates the hazard ratio and corresponding 95% CI, respectively, for each study. NE = not estimable.

**Figure 3:**
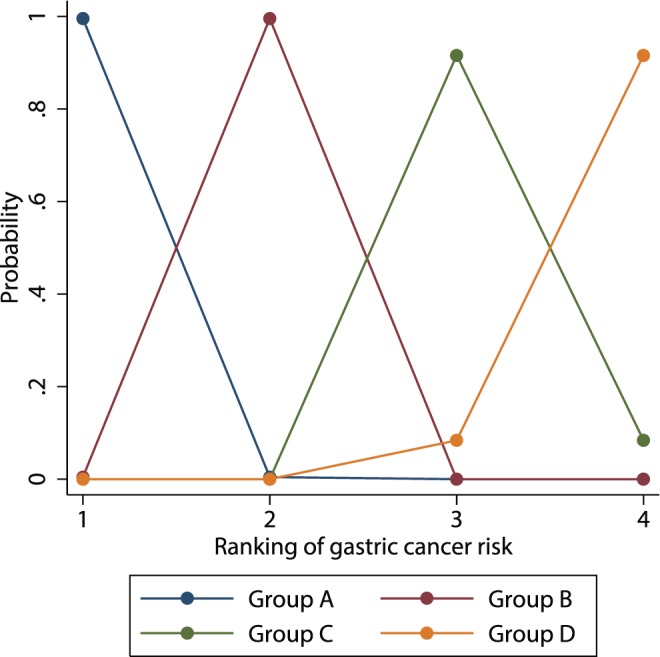
Rankogram of risk of gastric cancer development based on four-risk-group prediction model. Ranking probability of gastric cancer risk for each group, estimated from direct multivariate meta-analysis is shown. The 4 rankings show the risk of gastric cancer development: rank 1, lowest risk; rank 2, second lowest risk; rank 3, second highest risk; and rank 4, highest risk.

Four studies (a total of 16,943 subjects) that reported HRs [27], [35], [37], [39] were included in the meta-analysis of predictive ability. For predicting gastric cancer development, the 95% CrI of the summary HRs for 5 out of 6 possible contrasts did not include 1, suggesting that in the pairwise contrasts, other than the comparison between Group C and Group D, there was more than 95% probability that one of the two comparators had a higher risk of gastric cancer than the other (**Figure 2**). Specifically, multivariate meta-analyses suggested that Group A had a lower risk than Group B and Group C, and that compared with Group C and Group D, Group B had a lower risk. There was no significant difference in the risk of gastric cancer between Group C and Group D (summary HR, 1.49; 95% CrI: 0.84–2.65). The ranking analysis showed that Groups A and B, respectively, had the lowest and second-lowest risk of gastric cancer development (posterior cumulative probability to rank the lowest and the second-lowest risk groups was both >99%), whereas Groups C and D could be the highest or second-highest risk groups (92% and 8%, respectively, for being ranked as the second-highest group, and 8% and 92%, respectively, for the highest risk group) (**Figure 3**). In sensitivity analyses using alternative models, and subgroup analyses of only studies that adopted the recommended cutoff values for the pepsinogen test, the summary HR estimates as well as the results of the ranking analysis were similar to those of the main analysis (**Figure S4**).

Five studies (a total of 18,444 subjects) with cumulative count data [27], [35], [37], [39], [40] were included in the multivariate meta-analysis of OR. The summary estimates were similar to the findings in the meta-analysis of HR, and again, there was no evidence of difference between Group C and Group D (summary OR, 1.64; 95% CrI: 0.84–2.88) (**Figure S5**). The summary estimates for sensitivity analyses were stable and the results were not materially different from the main analysis (**Figure S6**). In the *post-hoc* sensitivity analysis of 3-risk-strata model, the multivariate meta-analysis and the ranking analysis showed that Group A had a lower risk than Group B and combined Group C and Group D, and compared with combined Group C and Group D, Group B had a lower risk (**Fig. S7–S9**).

**Figure 4:**
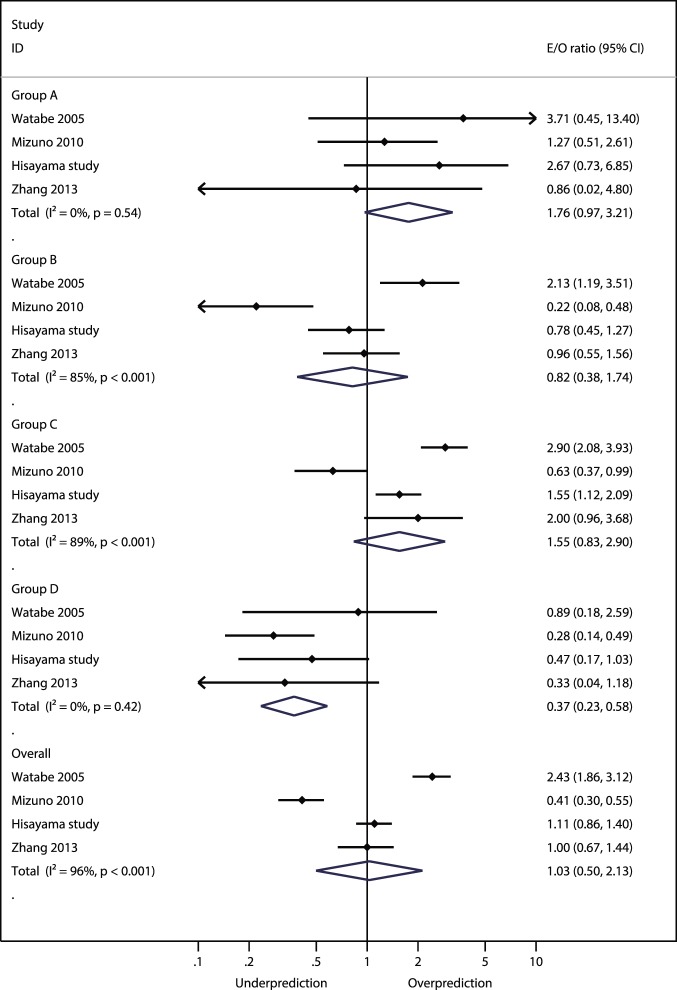
Meta-analysis of the expected over observed (E/O) ratios. The diamonds depict a summary E/O ratio and extending 95% confidence interval (CI). Each closed circle and horizontal line indicates the hazard ratio and corresponding 95% CI, respectively, for each study. Studies are ordered by publication year.

**Figure 5:**
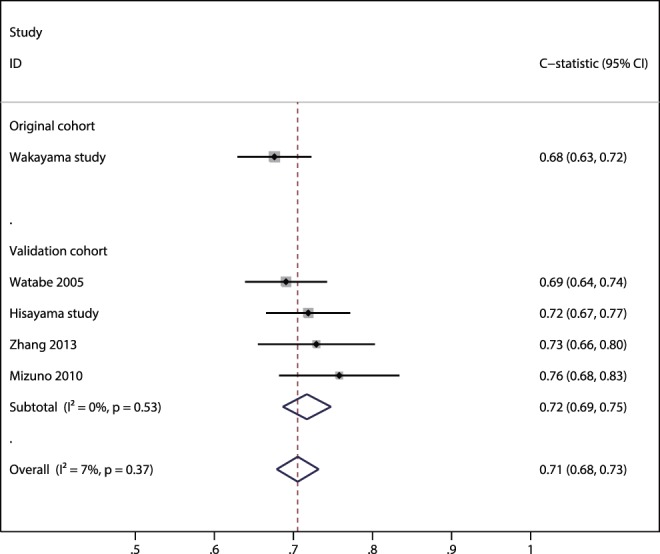
Meta-analysis of *c*-statistics. The diamonds depict a summary *c*-statistic and extending 95% confidence interval (CI). Each square and horizontal line indicates the hazard ratio and corresponding 95% CI, respectively, for each study. The size of each square is proportional to the weight of each study in the meta-analysis. Studies are ordered by sample size.

While two studies presented Kaplan-Meier plots of cumulative gastric cancer incidence by risk group [27], [35] and four studies calculated p-values for differences in gastric cancer incidence between the risk strata by Log-rank test [27], [35], [39] or Chi-squared test [40], none reported recommended statistical measures or graphical displays for assessing model performance of time-to-event data [22]. Although the meta-analysis for overall study population suggested that the calibration was generally good across all risk strata (summary E/O ratio, 1.03; 95% CI: 0.50–2.13; p = 0.94), high between-study heterogeneity was found (*I^2^* = 96%), suggesting that there were variations in the populations assessed in the validation studies (**Figure 4**). Specifically, the E/O ratio of one study showed an over-prediction (E/O, 2.43; 95% CI: 1.86–3.12; p<0.001), whereas an under-prediction was suggested for another study (E/O, 0.41; 95% CI: 0.30–0.55; p<0.001). In contrast, meta-analyses of the *c*-statistic suggested that the discrimination was in general fair with low evidence of between-study heterogeneity (summary *c*-statistic, 0.71; 95% CI: 0.68–0.73; *I^2^* = 7%) (**Figure 5**).

## Discussion

In this meta-analysis based on 9 prospective cohorts from Eastern Asia, we found that adults with a positive pepsinogen test, as a standalone test, had an approximately fourfold higher risk of gastric cancer than those with a negative test. Likewise, the risk of gastric cancer for those with positive *H. pylori* antibodies was about threefold higher than for those with a negative result. The performance of these tests did not seem to be different across the cohorts regardless of country or gastric cancer incidence. These findings are in general agreement with previous meta-analyses [9], [10], [43]–[46] based mostly on case-control and nested case-control studies, or cross-sectional studies.

In our multivariate meta-analysis, the prediction model seemed to be moderately accurate in separating asymptomatic adults into four risk groups. Although our results failed to show a significant difference between Group C and Group D, this should not be viewed as evidence that the risk of the two groups is equal because the lack of statistical significance may be due to small number of subjects categorized as Group D or events thereof.

Regarding the model performance, our descriptive meta-analysis found that the fair discriminatory performance reported from the first cohort seemed to be retained across the subsequent studies, whereas the calibration was not consistently validated, suggesting clinical heterogeneity across studies. One explanation could be that different screening settings enrolled different populations. Another might be variability in study design including different methodologies for diagnosing gastric cancer, follow-up time, and exclusion criteria adopted in the original studies.

Our study has several limitations. First, our meta-analysis is based on a small number of studies exclusively from Eastern Asia. Thus, our findings may not be generalizable to the populations in other regions. Second, our descriptive assessment of model performance is exploratory, based on the available cumulative count data with inconsistent follow-up periods and heterogeneous methods adopted to verify gastric cancer cases. Assessing how these affect the model performance would need data at the level of the individual. Third, the small number of eligible studies precluded subgroup analyses or meta-regression for *H. pylori* antibody assays. Therefore, how each different assay affects the results is unclear. Fourth, *H. pylori* and gastric atrophy are generally believed to be more relevant in the pathogenesis of intestinal type gastric cancer [7]. Few studies with the pertinent information precluded the subgroup analyses by histological subtype. Lastly, publication bias is still of concern because our searches were limited to the English- and Japanese-language literature.

Despite its development without formal statistical modeling and the paucity of rigorous external validation, the four-risk-group model has already been implemented in several screening programs including both private and public organizations in Japan. Given that the model is simple and both tests are easy to administer with minimal discomfort, the rapid acceptance is not surprising. A risk-stratified two-stage screening program incorporating the four-risk-group model may hold the promise of remedying the current low cancer screening rates; the risk model could efficiently select “high-risk” populations that would need a conventional screening modality while allowing those identified with a lower risk to omit the painful conventional tests. Notwithstanding these theoretical advantages, comparative evidence on clinically important outcomes such as improvements in gastric-cancer-specific mortality regarding the model-incorporated “stepwise” screening strategy compared with conventional strategies is still lacking and the consequences of withholding conventional screening tests from those labeled “low risk” by the model are unclear.

In summary, the serum pepsinogen test, *H. pylori* antibodies, and the four-risk-group prediction model seem to have the potential to stratify middle-aged presumptively healthy adults in Eastern Asia for predicting the risk of gastric cancer. Before wider implementation in daily practice, to understand how these two tests and the risk model in particular will affect clinically important outcomes of screened populations, future research needs to focus on comparative studies to evaluate the impact of screening programs adopting the risk model. Given the challenges in conducting randomized trials, a decision modeling analysis incorporating information on the risk model as well as data on effectiveness of therapeutic interventions would be a realistic first step to take [47]. However, even if the modeling analysis is positive, we should not automatically discard the possibility of generating randomized comparative evidence as in other cancer screening fields [48]. In addition, given the variable prevalence of *H. pylori* infection across different generations and different countries [49], and also the recent introduction of eradication therapies, both of which are expected to affect the test results, validation of the current model performance is still necessary [47].

## Supporting Information

Figure S1 **Quality assessment of studies included in the meta-analysis.** The stacked bar charts illustrate quality rating for risk of bias for predictive factor studies by the Quality In Prognosis Studies (QUIPS-2) tool (A) [16], and risk of bias (B) and concerns about applicability (C) for studies of both predictive factor and risk prediction model by the Prediction Study Risk of Bias Assessment tool (PROBAST) [17]. The percentages of studies that met the given ratings for each domain are shown.
(EPS)

Figure S2 **Meta-analysis of hazard ratio (A), odds ratio (B), and sensitivity and specificity (C) for the pepsinogen test to predict gastric cancer development.** The diamonds depict the summary hazard ratio (A) or odds ratio (B) and extending 95% confidence interval (CI). Each square and horizontal line indicates the hazard ratio and corresponding 95% CI, respectively, for each study. The size of the square is proportional to the weight of each study in the meta-analysis. Studies are ordered by sample size. Individual study estimates of sensitivity and specificity are plotted in the receiver operating characteristic (ROC) space (C). The size of each circle is proportional to the sample size for each study (all study participants). The dashed crescent boundary represents the 95% confidence region for the summary sensitivity and specificity (shown as the square). The solid line represents the summary ROC curve.
(EPS)

Figure S3 **Meta-analysis of hazard ratio (A), odds ratio (B), and sensitivity and specificity (C) for** ***H. pylori*** **antibodies to predict gastric cancer development.** The diamonds depict the summary hazard ratio (A) or odds ratio (B) and extending 95% confidence interval (CI). Each square and horizontal line indicates the hazard ratio and corresponding 95% CI, respectively, for each study. The size of the square is proportional to the weight of each study in the meta-analysis. Studies are ordered by sample size. Individual study estimates of sensitivity and specificity are plotted in the receiver operating characteristic (ROC) space (C). The size of each circle is proportional to the sample size for each study (all study participants). The dashed crescent boundary represents the 95% confidence region for the summary sensitivity and specificity (shown as the square). The solid line represents the summary ROC curve.
(EPS)

Figure S4 **Sensitivity analysis for multivariate meta-analysis of hazard ratio for the four-risk-group prediction model.** The red and blue diamonds and horizontal lines depict a summary hazard ratio and corresponding 95% credible interval (CrI), estimated from the fixed- or random-effects multivariate meta-analysis, respectively. Subgroup results for studies that adopted the conventional cutoff for pepsinogen levels are also shown.
(EPS)

Figure S5 **Meta-analysis of odds ratio for four-risk-group prediction model to predict gastric cancer development.** The red and blue diamonds depict a summary odds ratio with extending 95% confidence interval (CI) or 95% credible interval (CrI), estimated from direct meta-analysis or multivariate meta-analysis, respectively. Each square and horizontal line indicates the odds ratio and corresponding 95% CI, respectively, for each study.
(EPS)

Figure S6 **Sensitivity analysis for multivariate meta-analysis of odds ratio for the four-risk-group prediction model.** The red and blue diamonds and horizontal lines depict a summary odds ratio and corresponding 95% credible interval (CrI), estimated from the fixed- or random-effects multivariate meta-analysis, respectively. Subgroup results for studies that adopted the conventional cutoff for pepsinogen levels are also shown.
(EPS)

Figure S7 **Meta-analysis of odds ratio for three-risk-group prediction model to predict gastric cancer development.** The red and blue diamonds depict a summary odds ratio with extending 95% confidence interval (CI) or 95% credible interval (CrI), estimated from direct meta-analysis or multivariate meta-analysis, respectively. Each square and horizontal line indicates the hazard ratio and corresponding 95% CI, respectively, for each study.
(EPS)

Figure S8 **Sensitivity analysis for multivariate meta-analysis of odds ratio for the three-risk-group prediction model.** The red and blue diamonds and horizontal lines depict a summary odds ratio and corresponding 95% credible interval (CrI), estimated from the fixed- or random-effects multivariate meta-analysis, respectively. Subgroup results for studies that adopted the conventional cutoff for pepsinogen levels are also shown.
(EPS)

Figure S9 **Rankogram of risk of gastric cancer development based on three-risk-group prediction model.** Ranking probability of gastric cancer risk for each group, estimated from direct multivariate meta-analysis is shown. The 3 rankings show the risk of gastric cancer development: rank 1, lowest risk; rank 2, second lowest risk; rank 3, highest risk.
(EPS)

Table S1 **Test characteristics.**
(DOCX)

Table S2 **Quality assessment of included studies.**
(DOCX)

Checklist S1 **PRISMA Checklist.**
(DOC)
