# Supplementary material for: Prediction of Gastric Cancer Development by Serum Pepsinogen Test and Helicobacter pylori Seropositivity in Eastern Asians: A Systematic Review and Meta-Analysis
Source: PLoS One. 2014 Oct 14;9(10):e109783. doi: 10.1371/journal.pone.0109783 (PMC4196955; doi:10.1371/journal.pone.0109783)
Supplement: Table S1 — Test characteristics. (DOCX) [file pone.0109783.s010.docx]

Supplementary Table S1. Test characteristics.^a^

| **Study ID** | **Timing of sample analysis** | **Antibody for diagnosing *H. pylori* infection** |  |  |
| --- | --- | --- | --- | --- |
|  |  | **Assay** | **Positive criteria** | **Reported sensitivity/specificity, *%* (reference standard)** |
| Katsushika study **[31,32]** | At enrollment | NE | NE | NA |
| Wakayama study **[27,33]^b^** | At enrollment | Anti-*H. pylori* IgG antibody ELISA (MBL, Nagoya, Japan) | >50 U/mL for positive* | 94/93 (ND) |
| Watase 2004 **[34]** | At enrollment | NE | NE | NA |
| Watabe 2005 **[35]** | ND | ELISA GAP-IgG kit (Biomerica, CA, USA) | Per manufacture’ s protocol | 95/83 (culture) |
| Hisayama study **[36,37]^c^** | 14 years after enrollment | HM-CAP EIA kit (Enteric Products, NY, USA) | Per manufacture’ s instructions | ND |
| Kim 2008 **[38]** | ND | Genedia *H. Pylori* ELISA (Green Cross Medical Science, Gyeonggi, South Korea) | Per manufacture’ s instructions | 98/92 (ND) |
| Mizuno 2010 **[39]** | 7 years after enrollment | Pirikapalte G Helicobacter EIA (Biomerica, CA, USA) | 1+, 2+, and 3+ for positive | 100/95 (13C-urea breath test) |
| Zhang 2012 **[40]** | ND | Determiner *H. pylori* IgG antibody EIA kit (Enteric Products, Westbury, NJ, USA or Scimedix Corp., Denville, NJ, USA) | <1.8 for negative; 1.8-2.3 for questionable; >2.3 for positive | ND |
| Okuno 2012 **[41]** | At enrollment | NE | NE | NA |

^a^ All studies that reported the method used to measure pepsinogen concentrations [27,33,35-41] used an identical assay (RIA-Bead Kits (Dinabbot, Tokyo, Japan)) with a set of recommended cutoff values (pepsinogen I ≤ 70 ng/mL and pepsinogen I/II ≤ 3.0) to diagnose chronic atrophic gastritis. Compared with histologic assessment as the reference standard, a sensitivity of 71% and a specificity of 97% are reported with this criterion.

^b^ A criterion of PG I ≤ 70 ng/mL and PG I/II ≤ 3.0 as positive PG test, and >50 U/mL as positive *H. pylori* antibody was used as the main analysis. Additional criteria for subcategorization, PG I ≤ 70 ng/mL and PG I/II > 3.0, PG I > 70 ng/mL and PG I/II > 3.0,and PG I > 70 ng/mL and PG I/II ≤ 3.0 for subcategorizing Group B, PG I/II > 3.0 vs. PG I/II ≤ 3.0 for subcategorizing Group B and Group C; *H. pylori* antibody ≤500 U/mL vs. >500 U/mL for subcategorizing Group B and Group C; and PG I ≤ 30 ng/mL, 30 ng/mL < PG I ≤ 50 ng/mL, and PG I > 50 ng/mL for subcategorizing Group C were also used.

^c^ PG I ≤ 30 ng/mL and PG I/II ≤ 2.0 was also used as a “strong-positive” subgroup. Another cut-off values, PG I ≤ 59 ng/mL and PG I/II ≤ 3.9, estimated as the values for the maximum Youden’s index, were also estimated through an exploratory ROC analysis (sensitivity of 70% and specificity of 69% for predicting gastric cancer development, not for chronic atrophic gastritis) and used for the analysis of a 4-group risk model based on both PG test and *H. pylori* infection status.

EIA = enzyme immunoassay; ELIZA= enzyme-linked immunosorbent assay; Ig = Immunoglobulin; NA = not applicable; ND = no data; NE = not evaluated; PG = pepsinogen; RIA = radioimmunoassay;
