# Supplementary material for: Prediction of Gastric Cancer Development by Serum Pepsinogen Test and Helicobacter pylori Seropositivity in Eastern Asians: A Systematic Review and Meta-Analysis
Source: PLoS One. 2014 Oct 14;9(10):e109783. doi: 10.1371/journal.pone.0109783 (PMC4196955; doi:10.1371/journal.pone.0109783)
Supplement: Table S2 — Quality assessment of included studies. (DOCX) [file pone.0109783.s011.docx]

Supplementary Table S2. Quality assessment of included studies.

| **Study ID** | 1. **Primary vs. secondary analysis** | 1. **Selection of participants** | 1. **Description of tested population and inclusion and exclusion criteria** | 1. **Start of follow-up** | 1. **Test characteristics** | | | |
| --- | --- | --- | --- | --- | --- | --- | --- | --- |
|  |  |  |  |  | 1. Assay and positive criteria | 1. Blinding of test assessors to clinical outcomes | 1. Blinding of outcome assessors to test results | 1. Consideration of other standard risk factors |
| Katsushika study **[31,32]** | Primary | 20% (4490/21978) of all potentially eligible subjects | Population-based health checkup; no exclusion criteria | The date of the baseline health checkups (inferred) | Standard (inferred) | Unclear | Unclear | None |
| Wakayama study **[27,33]** | Primary | 81% (4655/5706) of all potentially eligible participants | Workplace health checkup; exclusion criteria described | The date of the baseline health checkups | Standard | Unclear | Unclear | Age, alcohol drinking, smoking |
| Watase 2004 **[34]** | Primary | 22% of all potentially eligible subjects | Population-based health checkup; exclusion criteria described | The date of the baseline health checkups | Standard (inferred) | Unclear | Unclear | None |
| Watabe 2005 **[35]** | Primary | 75% (6983/9293) of all potentially eligible subjects | Opportunistic health checkup; exclusion criteria described | The date of the baseline screening (inferred) | Standard | Unclear | Blinded | Age, gender |
| Hisayama study **[36,37]** | Secondary | 89% (2446/2742) of all potentially eligible subjects | Population-based health checkup; exclusion criteria described | The date of the health checkups (inferred) | Standard | Unclear | Unclear | Age, *H. Pylori* status, serum cholesterol levels, smoking, total intake energy, salt intake, vitamin B1 intake^a^ |
| Kim 2008 **[38]** | Primary | Unclear | Opportunistic; no exclusion criteria | The date of the baseline screening (inferred) | Standard | Unclear | Unclear | None |
| Mizuno 2010 **[39]** | Secondary | “Nearly 45%” | Population-based health checkup; no exclusion criteria | The date of the baseline health checkups (inferred) | Standard | Unclear | Blinded | Age, gender |
| Zhang 2012 **[40]** | Primary | 86% (1501/1741) of all potentially eligible subjects | Population-based health checkup; exclusion criteria described | The date of the baseline health checkups (inferred) | Standard | Unclear | Unclear | None |
| Okuno 2012 **[41]** | Primary | 97% (4383/4531) of all potentially eligible subjects | Workplace health checkup; exclusion criteria described | The date of the baseline health checkups (inferred) | Standard | Unclear | Unclear | Age, gender |

^a^ These factors were determined based on a stepwise selection.

Supplementary Table S2- continued.

| **Study ID** | 1. **Outcome ascertainment** | | 1. **Data analysis** | | | |
| --- | --- | --- | --- | --- | --- | --- |
|  | 1. Protocol (completeness) of follow-up | 1. Follow-up duration | 1. Predefined risk groups | 1. External and/or internal validation | 1. Consideration of established risk model | 1. Adjustment with other risk factors |
| Katsushika study **[31,32]** | Registry-based, not protocol-driven | <5 y | Predefined | None | NA | None |
| Wakayama study **[27,33]** | Annual barium radiography and PG test (ND) | 10-15 y | Predefined | None | NA | Age, alcohol drinking, smoking |
| Watase 2004 **[34]** | Registry-based, not protocol-driven | <5 y | Predefined | None | NA | None |
| Watabe 2005 **[35]** | Annual endoscopy (mean 5.1 times per follow-up (mean 4.7 y)) | <5 y | Predefined | None | NA | Age, gender |
| Hisayama study **[36,37]** | Registry-based, not protocol-driven; strict follow-up with fewer than 1% lost to follow-up | 10-15 y | Predefined^a^ | None | NA | Age, *H. Pylori* status, serum cholesterol levels, smoking, total intake energy, salt intake, vitamin B1 intake^b^ |
| Kim 2008 **[38]** | Annual to triennial endoscopy (ND) | 5-10 y | Predefined | None | NA | None |
| Mizuno 2010 **[39]** | Registry-based, not protocol-driven | 5-10 y | Unclear | None | NA | Age, gender |
| Zhang 2012 **[40]** | Annual home visit | 10-15 y | Predefined | None | NA | None |
| Okuno 2012 **[41]** | Self-report or physicians’ report confirmed with the information from the testing institutions, not protocol-driven | 10-15 y | Predefined | None | NA | Age, gender |

^a^ An exploratory analysis based on a best-case cutoff threshold was also performed.

^b^ These factors were determined based on a stepwise selection, not predetermined.

NA = not applicable.
